# Characteristics of the Cytotoxicity of *Taraxacum mongolicum* and *Taraxacum formosanum* in Human Breast Cancer Cells

**DOI:** 10.3390/ijms231911918

**Published:** 2022-10-07

**Authors:** Chien-Jung Lin, Jen-Tuo Chen, Lin-Jhen Yeh, Rong-Chi Yang, Shih-Ming Huang, Teng-Wei Chen

**Affiliations:** 1 Department of Chinese Medicine, Tri-Service General Hospital, National Defense Medical Center, Taipei City 114, Taiwan; 2 Department of Biochemistry, National Defense Medical Center, Taipei City 114, Taiwan; 3 School of Medicine, National Defense Medical Center, Taipei City 114, Taiwan; 4 Division of General Surgery, Department of Surgery, Tri-Service General Hospital, National Defense Medical Center, Taipei City 114, Taiwan

**Keywords:** triple-negative breast cancer, *Taraxacum*, cytotoxicity, oxygen consumption rate, ribotoxic stress

## Abstract

Breast cancer is a highly heterogeneous disease that has been clinically divided into three main subtypes: estrogen receptor (ER)- and progesterone receptor (PR)-positive, human epidermal growth factor receptor 2 (HER 2)-positive, and triple-negative breast cancer (TNBC). With its high metastatic potential and resistance to endocrine therapy, HER 2-targeted therapy, and chemotherapy, TNBC represents an enormous clinical challenge. The genus *Taraxacum* is used to treat breast cancer in traditional medicine. Here, we applied aqueous extracts from two Taraxacum species, *T. mongolicum* and *T. formosanum*, to compare their potential antitumor effects against three human breast cancer cell lines: MDA-MB-231 (ER^−^, PR^−^, and HER2^−^), ZR-75-1 (ER^+^, PR^+/−^, and HER2^−^), and MCF-7 (ER^+^, PR^+^, and HER2^−^). Our results show that *T. mongolicum* exerted cytotoxic effects against MDA-MB-231 cells, including the induction of apoptosis, the reduction of cell proliferation, the disruption of the mitochondrial membrane potential, and/or the downregulation of the oxygen consumption rate. Both *T. mongolicum* and *T. formosanum* decreased cell migration and colony formation in the three cell-lines and exerted suppressive effects on MCF-7 cell proliferation based on metabolic activity and BrdU incorporation, but an enhanced proliferation of ZR-75-1 cells based on BrdU incorporation. *T. formosanum* induced ribotoxic stress in MDA-MB-231and ZR-75-1 cells; *T. mongolicum* did not. In summary, these findings suggest that *T. mongolicum* showed greater cytotoxicity against all three tested breast cancer cell lines, especially the TNBC MDA-MB-231 cell line.

## 1. Introduction

About 2.2 million women around the world were diagnosed with breast cancer in 2020 [1]. Thanks to increased awareness, improved screening programs, and more advanced treatments, more women than ever are surviving breast cancer. However, there are large differences among countries in both the incidence of breast cancer and its mortality rate. Most cases can be attributed to pregnancy-related factors, hormone therapy, and/or lifestyle factors (i.e., alcohol intake, lack of exercise, a low-fiber diet, obesity, and smoking), among other risk factors [1].

Breast cancer is a heterogeneous disease that is clinically divided into three main subtypes: luminal estrogen receptor (ER)-positive and progesterone receptor (PR)-positive, which are further subdivided into luminal A and B; human epidermal growth factor receptor 2 (HER2)-positive; and triple-negative breast cancer (TNBC). Among them, TNBC, which accounts for 15% of all breast cancers and 25% of all breast-cancer-related deaths, is well known to have different clinical and biological characteristics than the other subtypes [2,3]. The expressions of ER, PR, and HER-2 are all eliminated, and the response to endocrine therapy and anti-HER-2 targeted therapy is poor, limiting treatment options compared with the other subtypes [2,4,5]. Given its high metastatic potential and low response to endocrine therapy or chemotherapy, TNBC presents a difficult clinical challenge. However, various candidate small-molecule drugs, including single-target drugs and repurposed drugs, have emerged as promising TNBC therapies [2,4]. The present findings suggest that multiple genes are involved in the different types of TNBC and that their expression profiles are disease-specific, making it difficult to develop a targeted therapy for all types of TNBC [2]. Herbal medicines are each a source of diverse molecules, and future research will focus on the characterization of the active components in medicinal herbs [6].

Natural products are a valuable source of life-saving drug molecules [7]. Their accumulated evolutionary adaptations and the novelty and diversity of their structures make them unparalleled in the development of new treatments. However, their natural scarcity, structural complexity, and unknown mechanisms of action are often barriers to further development in drug research. In addition, because of the absence of enzyme activity and/or small molecular binding sites, many biologically validated therapeutic targets, such as oncogenes or tumor suppressor genes, are considered chemically “undruggable” [8,9].

For centuries, the genus *Taraxacum*, commonly known as dandelion, has been used in traditional medicine for the relief and treatment of various diseases. Dandelion reportedly prevents liver fibrosis, inflammatory responses, oxidative stress, and diabetes, among others [10,11,12,13,14]. The identification of active compounds and working mechanisms against diseases could enable these herbal traditional medicines to emerge as commercial herbs. However, only a small proportion of dandelion species are currently being scientifically studied [10,11,14,15,16,17,18,19,20]. The genus *Taraxacum* has been used to treat breast cancer in traditional folk medicine [15,17,18,19,20,21]. Moreover, it was recently demonstrated that cell apoptosis related to the PERK/p-eIF2α/ATF4/CHOP endoplasmic reticulum stress axis [10] and suppression of IL-10/STAT3/PD-L1 signaling accounted for the anticancer effect of *T. mongolicum* ethanolic extract against TNBC [18], and the ethanolic extract also exhibited estrogenic activity [21]. In addition to species of dandelion, the detailed mechanisms of antitumor activities between ethanolic and aqueous extracts are valuable to address.

In the present study, we applied aqueous extracts from two species of *Taraxacum*, *T. mongolicum* and *T. formosanum*, to compare their potential antitumor effects against the MDA-MB-231 TNBC cell line and the ZR75-1 and MCF-7 hormone-dependent breast cancer cell lines.

## 2. Results

### 2.1. T. mongolicum Exhibited Cytotoxicity toward MDA-MB-231 Triple-Negative Breast Cancer Cells; T. formosanum Did Not

**Figure 1:**
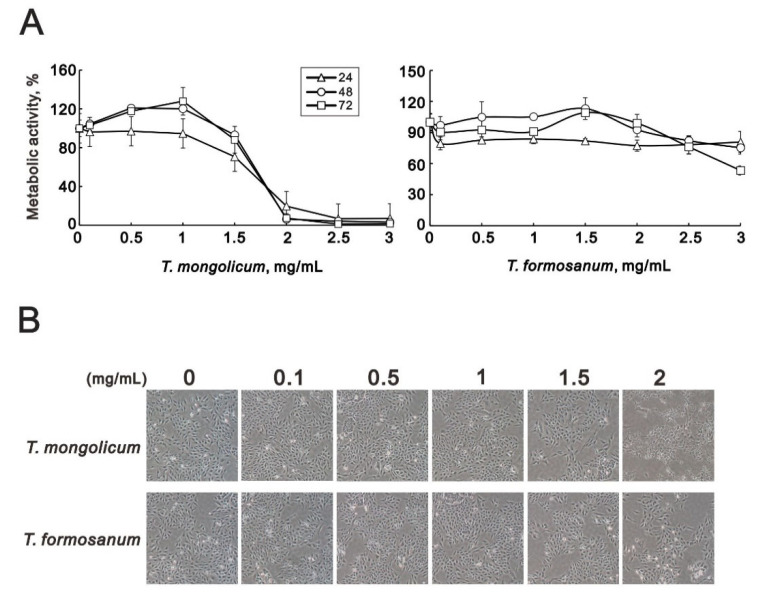
Cytotoxicity of *T. mongolicum* and *T. formosanum* in MDA-MB-231 cells. MDA-MB-231 cells were treated for 24, 48, or 72 h with the indicated concentrations of *T. mongolicum* and *T. formosanum*. (**A**) Metabolic activity was measured using MTT assays and (**B**) cell morphology was observed with light microscopy. The results are representative of three independent experiments. Symbols represent the mean ± SD.

We first tested the effect of extracts from *T. mongolicum* and *T. formosanum* on the metabolic activity of the MDA-MB-231 TNBC cells. The MTT assays showed that *T. mongolicum* decreased metabolic activities among MDA-MB-231 cells, and the effect was dose-dependent; *T. formosanum* did not (Figure 1A). We also observed that, at higher concentrations of *T. mongolicum,* the cells assumed a smaller size and irregular morphology (Figure 1B).

**Figure 2:**
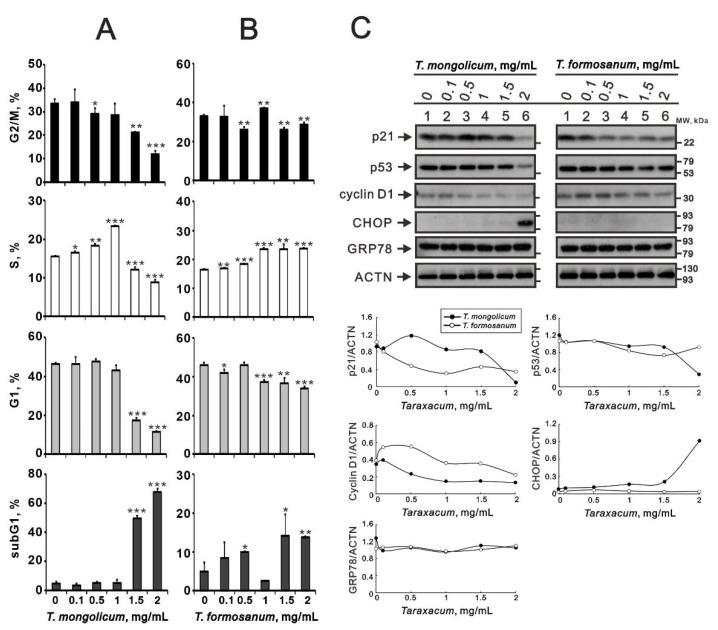
Cell cycle effects of *T. mongolicum* and *T. formosanum* on MDA-MB-231 cells. MDA-MB-231 cells were treated for 48 h with the indicated concentrations of (**A**) *T. mongolicum* or (**B**) *T. formosanum*. Cell cycle profiles were measured using flow cytometry after PI staining. The results were analyzed from three independent experiments. Symbols and bars depict the mean ± SD. * *p* < 0.05, ** *p* < 0.01, *** *p* < 0.001. (**C**) MDA-MB-231 cells were treated for 24 h with the indicated concentrations of *T. mongolicum* or *T. formosanum*. Cell lysates were subjected to Western blot analysis using antibodies against p21, p53, and cyclinD1. ACTN was the protein loading control. Protein bands were quantified through pixel density scanning and evaluated using ImageJ, version 1.44a (http://imagej.nih.gov/ij/) (accessed on 25 July 2022).

To investigate the mechanism(s) underlying *T. mongolicum* cytotoxicity, we next used cytometry for cell cycle analysis. We found that, at concentrations of 1.5 mg/mL or greater, *T. mongolicum* significantly induced subG1 populations while significantly decreasing the G1, S, and G2/M phase populations (Figure 2A). *T. formosanum* induced dose-dependent effects on the subG1 and S populations and decreased G1 populations (Figure 2B). Our previous data demonstrated that dose-dependent declines in these p53, cyclin D1, and p21 proteins were detected when cells were treated with *T. mongolicum* and *T. formosanum* in one human cervical cancer cell line, i.e., HeLa cells [16]. Analysis of the expression profiles of cell-cycle-related proteins, including p21, p53, and cyclin D1, revealed that *T. mongolicum* induced dose-dependent declines in the profiles of MDA-MB-231 cells (Figure 2C). Except for p53, *T.* *formosanum* induced dose-dependent declines in the profiles of p21 and cyclin D1 proteins. CHOP, one of the key unfolded protein response proapoptotic players, was positively transcribed by the PERK–eIF2–ATF4 axis for endoplasmic reticulum stress [22,23]. We observed the induction of CHOP by *T. mongolicum* in MDA-MB-231 cells, which was similar to our previous study [16]; *T. formosanum* did not induce CHOP. The GRP78 protein, an endoplasmic reticulum stress sensor protein, remained at a constant level.

**Figure 3:**
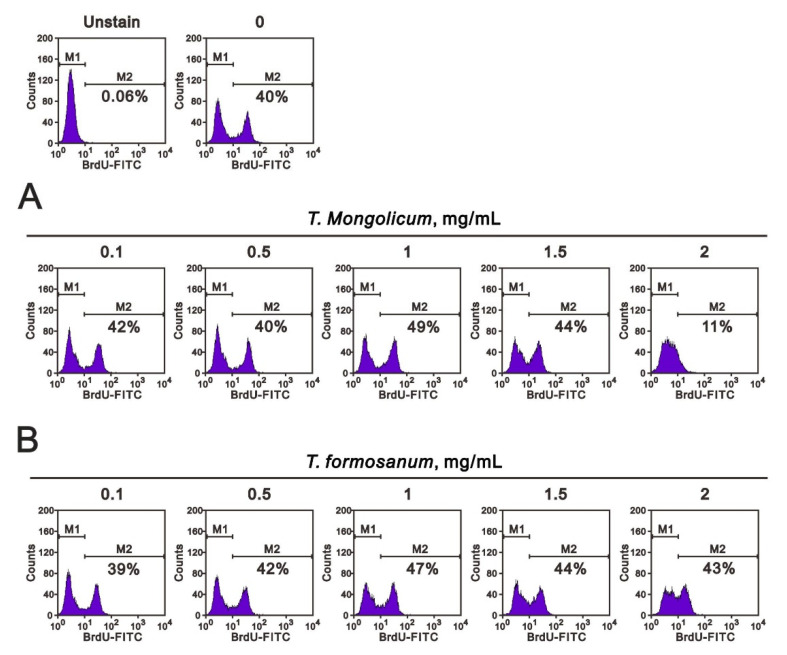
Effects of *T. mongolicum* and *T. formosanum* on MDA-MB-231 cell proliferation. MDA-MB-231 cells were treated for 48 h with the indicated concentrations of (**A**) *T. mongolicum* or (**B**) *T. formosanum*. They were then subjected to BrdU proliferation analysis.

Given the observed decrease in the S phase population induced by *T. mongolicum* and the mild increase in that population induced by *T. formosanum*, we further analyzed the effects of the two extracts on MDA-MB-231 cell growth by performing BrdU and colony formation analyses. In the BrdU analysis, the cell proliferation rate was significantly decreased from 40% to 11% by *T. mongolicum* at 2 mg/mL, whereas the rate was increased from 39% to 43% by *T. formosanum* (Figure 3). Notably, however, at a concentration of 1 mg/mL, *T. mongolicum* increased the proliferation rate from 40% to 49%, while *T. formosanum* increased it from 40% to 47%.

**Figure 4:**
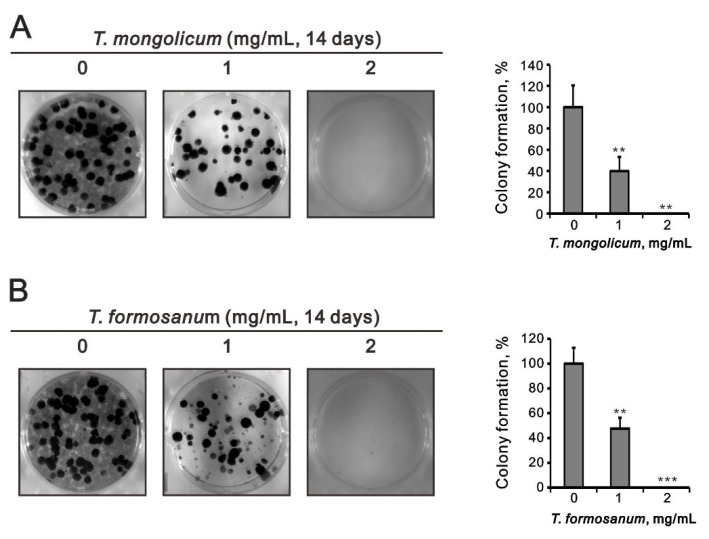
Effects of *T. mongolicum* and *T. formosanum* on colony formation by MDA-MB-231 cells. MDA-MB-231 cells were treated for 14 days with the indicated concentrations of (**A**) *T. mongolicum* or (**B**) *T. formosanum*. They were then subjected to colony formation analysis. The results are representative of three independent experiments. Bars depict the mean ± SD. ** *p* < 0.01. *** *p* < 0.001.

In subsequent colony formation assays, we counted the numbers of cell colonies formed over 14 days in culture. It was found that at 1 mg/mL both *T. mongolicum* and *T. formosanum* significantly decreased colony formation and completely blocked colony formation at 2 mg/mL (Figure 4).

**Figure 5:**
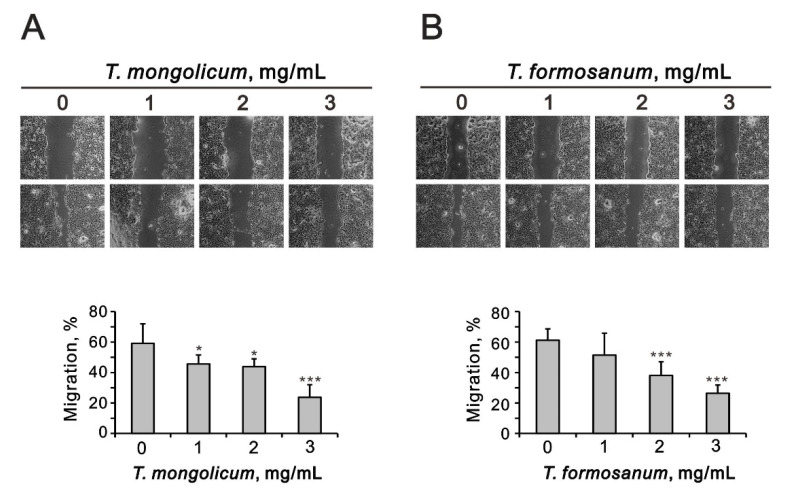
Effect of *T. mongolicum* and *T. formosanum* on MDA-MB-231 cell migration. MDA-MB-231 cells were treated with the indicated concentrations of (**A**) *T. mongolicum* or (**B**) *T. formosanum*. After 20 h, the cell migration was measured. The results are representative of two independent experiments. Bars depict the mean ± SD. * *p* < 0.05, *** *p* < 0.001.

In monolayer wound assays, *T. mongolicum* and *T. formosanum* significantly decreased MDA-MB-231 cell migration (Figure 5A,B). Our data show that the suppressive effect of *T. mongolicum* was better than that for *T. formosanum*.

**Figure 6:**
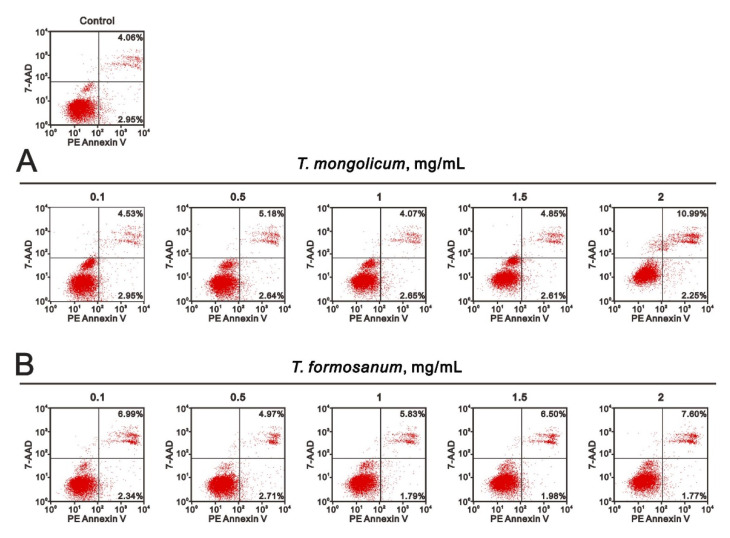
Effects of *T. mongolicum* and *T. formosanum* on apoptosis in MDA-MB-231 cells. MDA-MB-231 cells were treated for 24 h with the indicated concentrations of (**A**) *T. mongolicum* or (**B**) *T. formosanum*. They were then subjected to Annexin V apoptosis analysis. Early apoptotic cells are PE Annexin V-positive and 7-AAD-negative, while late apoptotic cells are both PE Annexin V-positive and 7-AAD-positive.

The increase in subG1 populations suggests that *T. mongolicum* cytotoxicity might involve the induction of apoptosis. To test that idea, we used Annexin V and 7-AAD kits to assess the induction of apoptosis and necrosis by *T. mongolicum*. Annexin V is a member of the annexin family of intracellular proteins that binds to phosphatidylserine (PS) in a calcium-dependent manner. In healthy cells, PS is only distributed in the inner leaflet of the cell membrane lipid bilayer; however, during early apoptosis, membrane asymmetry is lost, and PS is translocated to the outer leaflet, where Annexin V binds to it, differentiating the healthy cells from the apoptotic ones. The cells were also exposed to 7-AAD solution to help distinguish apoptotic from necrotic cells. Early apoptotic cells exclude 7-AAD, while late apoptotic or necrotic cells do not, enabling the dye to pass into the nucleus and bind to DNA. At a concentration of 2 mg/mL, *T. mongolicum* increased the MDA-MB-231 cell population in late apoptosis (Figure 6); *T. formosanum* did not.

### 2.2. Effects of T. mongolicum and T. formosanum on Mitochondrial Membrane Potential, Respiration, and Glycolysis in MDA-MB-231 Cells

**Figure 7:**
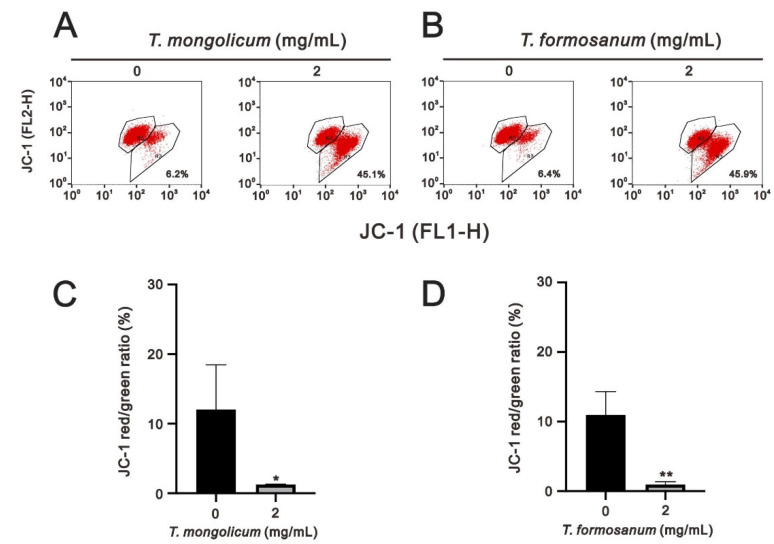
Effects of *T. mongolicum* and *T. formosanum* on mitochondrial membrane potential in MDA-MB-231 cells. MDA-MB-231 cells were treated with 2 mg/mL of (**A**) *T. mongolicum* or (**B**) *T. formosanum* for 24 h. Mitochondrial depolarization was measured as a function of a decrease in the red/green (FL2-H/FL1-H) fluorescence intensity ratio. (**C**,**D**) are the results of three independent experiments. Symbols and bars depict the mean ± SD. * *p* < 0.05, ** *p* < 0.01.

Mitochondrial status plays a central role in the control of cell life and death [24]. The effects can lead to cell death via the disruption of electron transport, oxidative phosphorylation, and adenosine triphosphate (ATP) production. One consequence of the loss of electron transport is the reduced production of ATP. While a decline in ATP production is observed during apoptosis, it often occurs relatively late in the process. However, the mitochondrial membrane potential is a key indicator of mitochondrial activity, as it reflects the driving force of ATP production, including the process of electron transfer and oxidative phosphorylation. Therefore, we first used voltage-sensitive JC-1 dye to monitor the changes in mitochondrial membrane potential. The monomeric form (green fluorescence) of JC-1 indicates a loss of mitochondrial membrane potential compared with the normal aggregated form (red fluorescence). We observed that both *T. mongolicum* and *T. formosanum* dose-dependently disrupted mitochondrial membrane potential. We further analyzed the highest dosage (2 mg/mL) of *T. formosanum* and *T. mongolicum* on changes in mitochondrial membrane potential (Figure 7, the ratio of red/green). Both *T. formosanum* and *T. mongolicum* significantly disrupted the mitochondrial membrane potential (the decline of the red/green ratio) of MDA-MB-231 cells (*p* = 0.022 for *T. mongolicum* and *p* = 0.003 for *T. formosanum*).

**Figure 8:**
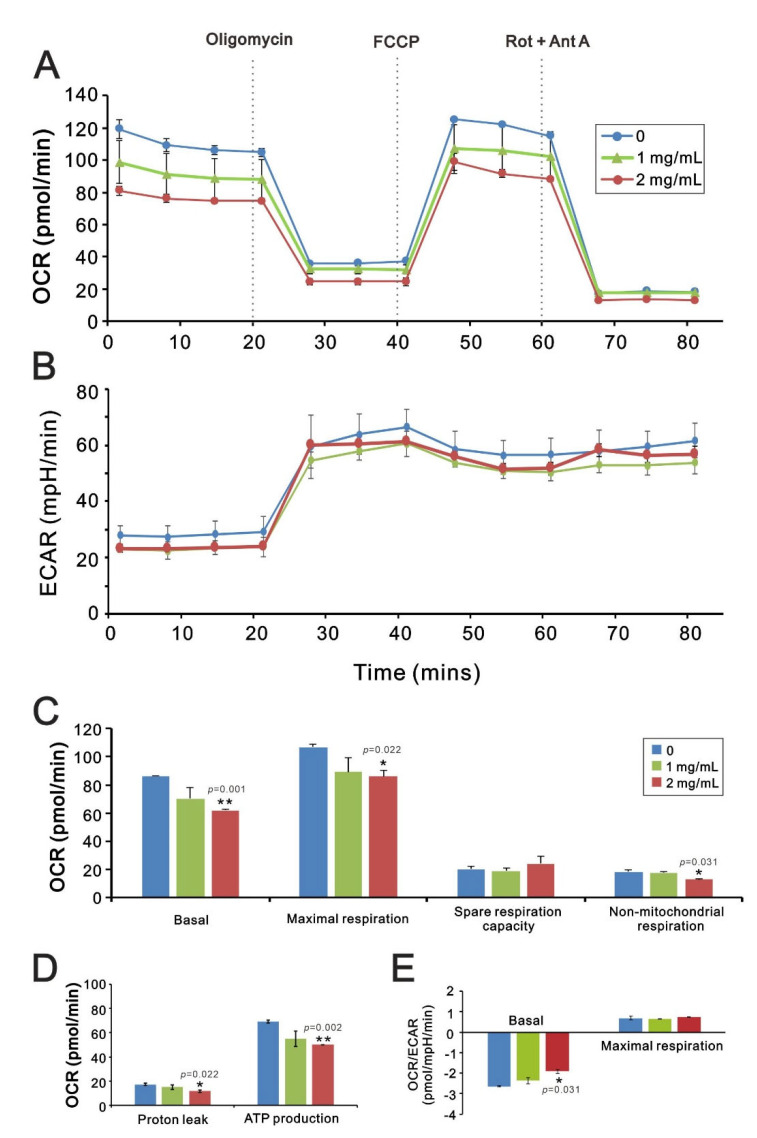
Effects of *T. mongolicum* on the oxygen consumption rate (OCR) and extracellular acidification rate (ECAR) in MDA-MB-231 cells. (**A**–**D**) MDA-MB-231 cells were treated for 24 h with the indicated concentrations of *T. mongolicum*, after which cellular OCR (**A**,**C**,**D**), ECAR (**B**), and the OCR/ECAR ratio (**E**) were measured in XF24 bioenergetic assays. Symbols and bars depict the mean ± SD. * *p* < 0.05, ** *p* < 0.01.

We then analyzed the effects of *T. mongolicum* on mitochondrial respiration, glycolysis, and ATP production in MDA-MB-231 cells based on measurements of the oxygen consumption rate (OCR) and extracellular acidification rate (ECAR) using a Seahorse XF analyzer. Our results show that *T. mongolicum* dose-dependently decreased the OCR but not the ECAR (Figure 8A,B), which suggests its primary effect is on mitochondrial respiration, not glycolysis. At a concentration of 2 mg/mL, *T. mongolicum* significantly decreased basal respiration, maximal respiration, non-mitochondrial respiration, proton leakage, and ATP production (Figure 8C,D, *p* = 0.001, 0.022, 0.031, 0.022, and 0.002, comparing 2 mg/mL *T. mongolicum* with vehicle alone). Correspondingly, 2 mg/mL *T. mongolicum* also decreased OCR/ECAR ratios during basal respiration; however, it did not during maximal respiration (Figure 8E, *p* = 0.031, comparing 2 mg/mL *T. mongolicum* with vehicle alone).

### 2.3. The Ribotoxic Stress Response of T. mongolicum and T. formosanum on MDA-MB-231 Cells

**Figure 9:**
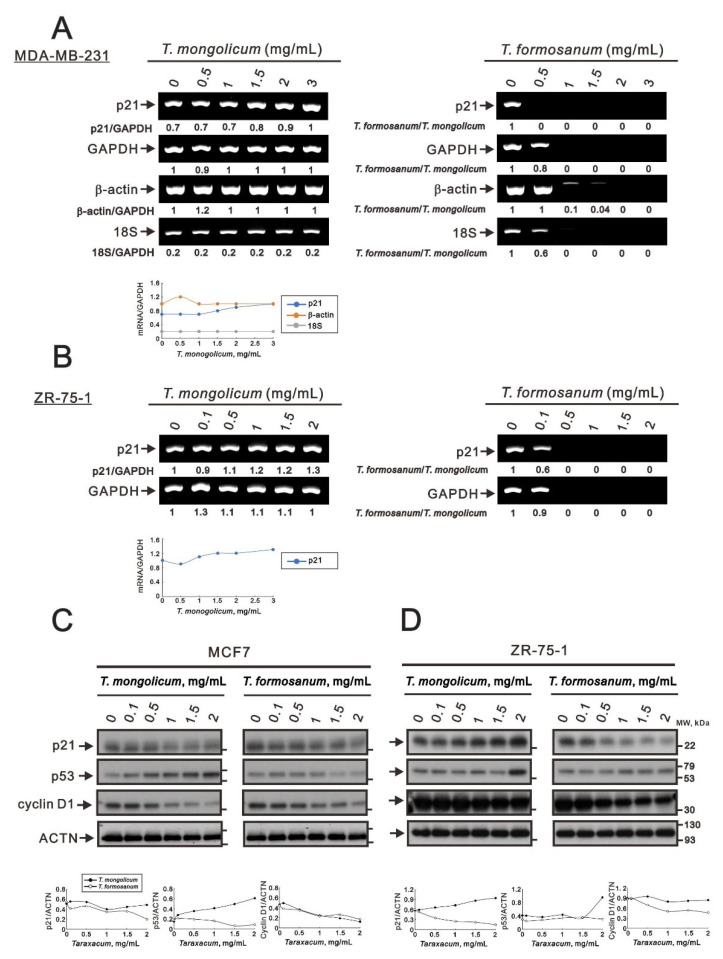
Effects of *T. mongolicum* and *T. formosanum* on transcriptional regulation in MDA-MB-231 cells. (**A**) MDA-MB-231 and (**B**) ZR-75-1 cells were treated for 24 h with the indicated concentrations of *T. mongolicum* or *T. formosanum*, after which their cell lysates were subjected to RT-PCR analysis using primer pairs against *p21* mRNA, with *GAPDH* and *β-actin* mRNAs serving as mRNA loading controls and 18S rRNA as an internal control. (**C,D**) Western blot analysis using antibodies against the indicated proteins, with ACTN serving as the protein loading control. mRNA and protein bands were quantified through pixel density scanning and evaluated using ImageJ, version 1.44a (http://imagej.nih.gov/ij/) (accessed on 25 July 2022). The values of the *GAPDH* mRNA of *T. mongolicum* were calculated with the vehicle only, and other values were calculated as the indicated ratio.

Our previous study suggested that *T. mongolicum* and *T. formosanum* may decrease ribosomal RNA synthesis via the ribotoxic stress response in human cervical cancer cells [16]. *Taraxacum* decreased cellular levels of total RNA with the cleavage of 28S rRNA and *GAPDH* and *β-actin* mRNAs, but not proteins, in HeLa cells. Based on the ethanolic extract of *T. mongolicum* exhibiting estrogenic activity in MCF-7 (ER^+^, PR^+^, and HER2^−^) cells [21], we examined the functional role of ER for the ribotoxic stress response in MDA-MB-231 (ER^−^, PR^−^, and HER2^−^) and ZR-75-1 (ER^+^, PR^+/−^, and HER2^−^) cells. We found that in both MDA-MB-231 and ZR-75-1 cells, *T. formosanum,* at concentrations of 1 mg/mL or greater, suppressed the expression of *p21* and *GAPDH* mRNAs; *T. mongolicum* had no decreased effect on any internal control (*GAPDH* or *β-actin* mRNA or 18s rRNA) and induced expression of *p21* mRNA in both cell types (Figure 9A,B). *T. formosanum* also decreased or further suppressed the expression of another two internal controls, *β-actin* mRNA and 18S rRNA, in MDA-MB-231 cells (Figure 9A,B), suggesting similar effects on mRNA and proteins to HeLa cells [16]. Based on the suppression of some internal controls with higher dosages, the ribotoxic stress effects were measured using the ratio of *T. formosanum*/*T. mongolicum*. The ratio value of *T. formosanum*/*T. mongolicum*, including internal controls or *p21*, was closer to zero, suggesting that *T. formosanum* induced the ribotoxic effect in both MDA-MB-231 and ZR-75-1 cells.

The effect of *T. mongolicum* on p21 protein expression was reduced in MCF-7 cells; in contrast, it was induced in ZR-75-1 cells. The expression of p21 protein was decreased by *T. formosanum* in both MCF-7 and ZR-75-1 cells. Our data show that the effect of *T. mongolicum* on p21 protein was consistent with its mRNA in ZR-75-1 but not MDA-MB-231 cells (compare Figure 2C and Figure 9B). The effect of *T. formosanum* on p21 protein was inconsistent with the mRNA of ZR-75-1 and MDA-MB-231 cells; however, it was similar to the findings of GAPDH [16]. *T. mongolicum* elevated the p53 protein expression in MCF-7 and ZR-75-1 cells. The p53 protein expression was decreased by *T. formosanum* in MCF-7 cells; however, it was increased in ZR-75-1 cells. The suppressive effect on cyclin D1 protein expression by *T. mongolicum* and *T. formosanum* was observed in MCF-7 and ZR-75-1 cells (Figure 9C,D), suggesting that the aqueous extracts of *T. mongolicum* and *T. formosanum* might not have estrogenic activity.

### 2.4. Comparison of the Effects of T. mongolicum and T. formosanum on Cell Proliferation, Migration, and Colony Formation in Three Breast Cancer Cell Lines

**Figure 10:**
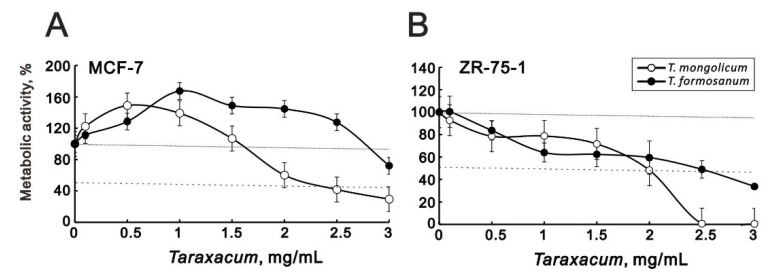
Cytotoxicity of *T. mongolicum* and *T. formosanum* toward MCF-7 and ZR-75-1 cells. (**A**) MCF-7 and (**B**) ZR-75-1 cells were treated for 72 h with the indicated concentrations of *T. mongolicum* or *T. formosanum*. Cell viability was measured using MTT assays, and (**B**) cell morphology was observed using light microscopy. The results are representative of three independent experiments. Symbols depict the mean ± SD.

Given that the cytotoxicity toward MDA-MB-231 cells differed between *T. mongolicum* and *T. formosanum* (Figure 1), we also compared their cytotoxic effects against MCF-7 and ZR-75-1 hormone-dependent cells. Our metabolic activity analysis showed that *T. formosanum* only modestly decreased MCF-7 metabolic activity at a concentration of 3 mg/mL. *T. mongolicum* dose-dependently suppressed the metabolic activity of these cells at concentrations greater than 1.5 mg/mL (Figure 10A). In ZR-75-1 cells, both *T. mongolicum* and *T. formosanum* decreased metabolic activity, with *T. mongolicum* completely suppressing metabolic activity at 2.5 mg/mL and higher (Figure 10B).

**Figure 11:**
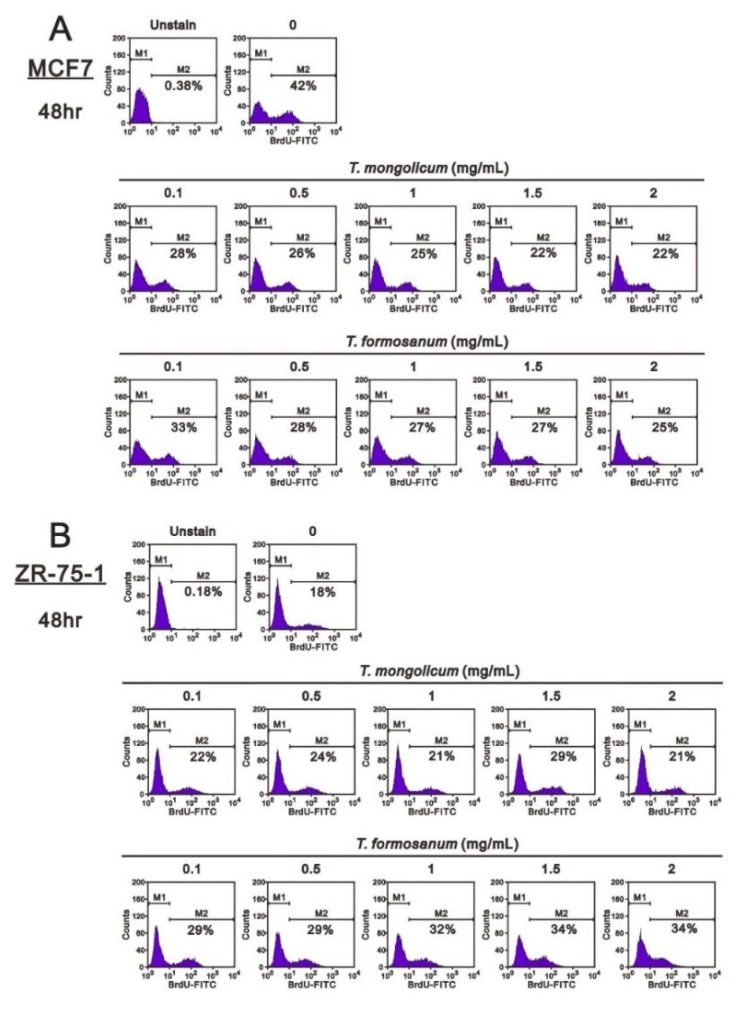
Effects of *T. mongolicum* and *T. formosanum* on cell proliferation in MCF-7 and ZR-75-1 cells. (**A**) MCF-7 and (**B**) ZR-75-1 cells were treated for 48 h with the indicated concentrations of *T. mongolicum* or *T. formosanum*. They were then subjected to BrdU proliferation analysis.

Figure 3 shows that MDA-MB-231 cell proliferation was suppressed by 2 mg/mL *T. mongolicum*; however, it was enhanced by *T. formosanum*. Both *T. mongolicum* and *T. formosanum* decreased the MCF-7 cell proliferation (Figure 11A); however, they enhanced the proliferation of ZR-75-1 cells based on BrdU incorporation (Figure 11B).

**Figure 12:**
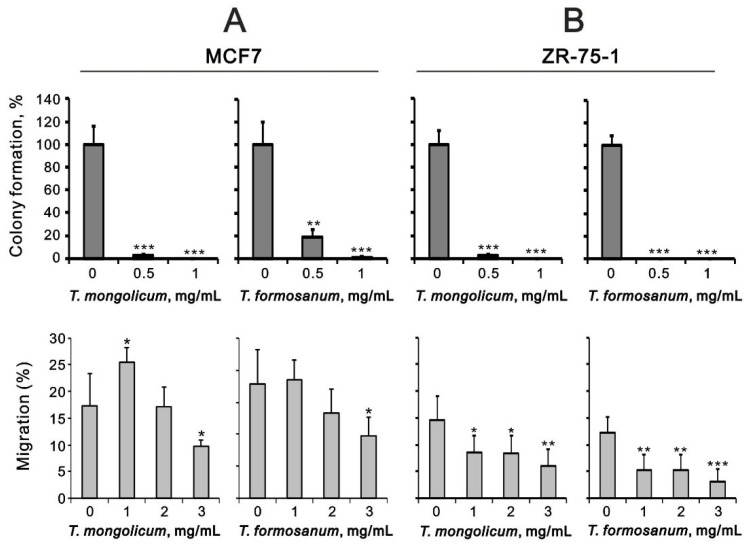
Effect of *T. mongolicum* and *T. formosanum* on colony formation by MCF-7 and ZR-75-1 cells. (**A**) MCF-7 and (**B**) ZR-75-1 cells were treated with the indicated concentrations of *T. mongolicum* or *T. formosanum*. Colony formation (**top**) was measured after 14 days, and cell migration (**bottom**) was measured after 24 h. The results are representative of two independent experiments. Bars depict the mean ± SD. * *p* < 0.05, ** *p* < 0.01, *** *p* < 0.001.

In MCF-7 and ZR-75-1 cells, *T. mongolicum* and *T. formosanum* both significantly decreased colony formation (Figure 12) and cell migration in a dose-dependent manner (Figure 12). The suppressive effects of cell migration by *T. mongolicum* and *T. formosanum* were better in ZR-75-1 cells than in MCF-7 cells.

## 3. Discussion

Because TNBC tumors lack expression of the ER, PR, and HER-2 proteins, their response to endocrine therapy and anti-HER-2 targeted therapy is poor [2,3,4]. The variety of health benefits associated with the use of dandelions has been attributed to certain species of *Taraxacum* prepared as extracts of entire plants or specific plant parts. Here, we observed that *T. mongolicum* exerted a better cytotoxic effect than *T. formosanum* against the MDA-MB-231 TNBC cell line. Moreover, the results demonstrate that cytotoxicity was mediated through the induction of apoptosis, suppression of cell proliferation, disruption of the mitochondrial membrane potential, and/or downregulation of the OCR. Both *T. mongolicum* and *T. formosanum* decreased cell migration and colony formation in MDA-MB-231, MCF-7, and ZR-75-1 cells and exerted suppressive effects on MCF-7 cell proliferation; however, they enhanced the proliferation of ZR-75-1 cells. *T. formosanum* induced ribotoxic stress in MDA-MB-231 and ZR-75-1 cells; *T. mongolicum* did not. Taken together, these findings suggest *T. mongolicum* showed better cytotoxicity against all three tested breast cancer cell lines, especially the MDA-MB-231 TNBC cells. The primary cytotoxicity resulted from the induction of apoptosis and the suppression of cell proliferation.

*T. officinale* is distributed worldwide and is the subject of more than 70% of pharmacological references to *Taraxacum*. Phytochemical investigations have revealed that dandelion contains chicoric acid, flavonoids, lactones, phenolics, polysaccharides, sesquiterpenes, and triterpenes [10,11,14,15,17,20]. Importantly, several bioactive compounds extracted from various *Taraxacum* species, including *T. mongolicum* and *T. formosanum*, remain to be identified. In contrast to HeLa cells [16], the effect of *T. mongolicum* on cytotoxicity was better than that of *T. formosanum* in human breast cancer cells in this study. No common receptor (ER, PR, or HER2) target and multiple genes are involved in the different types of TNBC, making it difficult to develop a targeted therapy for all types of TNBC [2]. Endocrine therapy via suppression of estrogen production and/or ER targets for ER-positive (ER^+^) breast cancer has considerably reduced the recurrence of and the mortality from breast cancer. However, de novo and acquired resistance to this treatment remain a major challenge [5]. Here, we examined and observed the cytotoxic effect of aqueous extracts from *T. mongolicum* and *T. formosanum* in MDA-MB-231 (ER^−^, PR^−^, and HER2^−^), ZR-75-1 (ER^+^, PR^+/−^, and HER2^−^), and MCF-7 (ER^+^, PR^+^, and HER2^−^) cell lines.

The ethanolic extract of *T. mongolicum* exhibited estrogenic activity in MCF-7 cells, supporting the application of *Taraxacum* in herbal medicines [21]. Cyclin D1 is one of the target genes of ER [25], but its protein and/or mRNA were suppressed in HeLa [16], MDA-MB-231, and ZR-75-1 cells (this study). Recently, many papers have focused on the working mechanisms of *Taraxacum* in human breast cancer cells [15,16,17,18,19,20,21]. The anticancer effect of an ethanolic extract from *T. mongolicum* against TNBC was reported [18]. It appears that apoptosis, related to signaling by the endoplasmic reticulum stress PERK/p-eIF2α/ATF4/CHOP axis, accounts for the anticancer effect. Most of the identified compounds in the extract were flavonoids and phenolic acids, which, based on earlier studies, the authors suggested may be responsible for the anticancer activity of dandelion extract [18,19]. CHOP was verified to be involved in the endoplasmic reticulum stress cell death [18], but its overexpression is not sufficient to kill cells [26]. In addition, CHOP is one of the transcription factors for genes involved in the pro-survival autophagic process [27]. Hence, CHOP plays a pivotal role in switching between autophagy and apoptosis. In this study, we applied aqueous extracts from *T. mongolicum* and *T. formosanum*. Only *T. mongolicum* induced CHOP protein expression in MDA-MB-231 cells, suggesting that the aqueous extract of *T. mongolicum* may be effective via CHOP-dependent apoptosis, as the ethanolic extract was. Dr. Qu’s laboratory recently demonstrated the multitarget mechanisms of *T. mongolicum* against TNBC using network pharmacology, molecular pharmacology, and metabolomics approaches [15], which provide multiple platforms by which to identify the potential bioactive compound(s) and working mechanisms for the *Taraxacum* study. Hence, we may address the issue of whether CHOP is essential for endoplasmic reticulum stress-induced apoptosis and/or autophagy through the use of bioactive compound(s) of *T. mongolicum*.

The ribotoxic stress response can sense stress in highly conserved regions of the 28S rRNA via the activation of mitogen-activated protein kinases [28]. Ribosomes containing the cleaved 28S rRNA may be unable to form polysomes or may be functionally inactive for protein synthesis [29], mediated through the activation of cellular endoribonuclease RNase L [30], and/or apoptosis-associated rRNA cleavage [31]. Ribotoxic stress is sensed by the ZAK, leading to the activation of p38 and JNK and inflammatory signaling. In the case of strong and sustained signaling, the cell will undergo regulated cell death, which can be used in anticancer drugs, such as doxorubicin. This signaling may be linked to some of the detrimental side effects of chemotherapy-related cardiotoxicity [29,32]. Our previous study demonstrated that *T. formosanum* and *T. mongolicum* exerted ribotoxic stress in Hep3B cells; however, only *T. formosanum* exerted ribotoxic stress in HepG2 cells, whereas *T. mongolicum* did not [16]. Similar to the effect on HepG2 cells, only *T. formosanum* exerted ribotoxic stress in MDA-MB-231 and ZR-75-1 cells in the current study; *T. mongolicum* did not. Higher concentrations of *T. mongolicum* were able to induce a ribotoxic stress response in HeLa cells, suggesting that the optimal concentration of *T. mongolicum* might have the ability to induce the ribotoxic stress response in human breast cancer cells. However, the detailed mechanisms and functions of the ribotoxic stress response, and whether it is involved in the cytotoxicity of cancer cells by *Taraxacum,* remain to be further investigated. Compared with many known ribotoxic stress agents, it should be an interesting task to identify the potential bioactive compound(s) from endemic *T. formosanum* aqueous extracts.

The selectivity and differential efficacy of *T. formosanum* and *T. mongolicum* on several cancer cells were demonstrated in our collaborating laboratories. In human cervical cancer cells, *T. formosanum* more strongly induced apoptosis and endoplasmic reticulum stress and suppressed cellular proliferation, transcription, colony formation, migration, and invasion than *T. mongolicum* [16]. In human breast cancer cells, *T. mongolicum* exerted better cytotoxic effects than *T. formosanum* mediated through the induction of apoptosis, the reduction of cell proliferation, the disruption of the mitochondrial membrane potential, and/or the downregulation of the oxygen consumption rate. In Huh6 liver cancer cells the induction effect of *T. mongolicum* on the subG1 population was stronger than that of *T. formosanum* [16]. The knowledge gap of the above-mentioned findings not only comprises the detailed phytochemical information of various species of *Taraxacum*, but also includes efficient systems and pharmacology platforms containing ideal information convergence of working targets, associated molecules, and functional interaction networks. Therefore, future research will focus on the characterization of the active components of *Taraxacum* and the effects of various combinations for therapeutic advancement and pharmaceutical product development.

## 4. Materials and Methods

### 4.1. Preparation of Aqueous Taraxacum Extracts

*T. mongolicum* was purchased from the Yu Hong Biotechnology Company, Chiayi, Taiwan, ROC (batch number: YF108011186), and *T. formosanum* was identified and provided by Dr. Rong-Chi Yang, Adjunct Associate Professor, School of Traditional Chinese Medicine, Chang Gung University (Taoyuan, Taiwan, ROC). All aqueous *Taraxacum* extracts were prepared and dissolved in boiled ddH_2_O to the desired final concentration (mg/mL) for cell treatment, as previously described [16].

### 4.2. Cell Culture

MDA-MB-231 human TNBC cells and ZR75-1 and MCF-7 non-TNBC human breast cancer cells were cultured in Dulbecco’s modified Eagle’s medium (DMEM), containing 10% fetal bovine serum (FBS) and 1% penicillin-streptomycin (Invitrogen, Waltham, MA, USA). 

### 4.3. Metabolic Activity Analysis

Cells were plated into 24-well culture plates and incubated for 1 day, after which fresh DMEM containing the indicated drugs was added to each well. The procedural details were described in our previous publications [33,34]. The cells were incubated with these treatments for the indicated periods. Then, the cells were incubated in MTT solution for 1 h at 37 °C. After adding dimethyl sulfoxide (DMSO; 200 μL), the absorbances at 570 nm and 650 nm were measured using an ELISA plate reader (Multiskan EX, Thermo, Waltham, MA, USA). The metabolic activity was calculated based on the absorbance ratio between the cells cultured with the indicated drugs and the untreated controls, which were assigned a value of 100.

### 4.4. Western Blot Analysis

Cells were lysed in radio-immunoprecipitation assay buffer (100 mM Tris-HCl pH 8.0, 150 mM NaCl, 0.1% SDS, and 1% Triton X-100) at 4 °C. Protein concentrations in the lysates were measured using a DC Protein Assay Kit (Bio-Rad Laboratories, Hercules, CA, USA). Proteins in aliquots of the lysate were separated by 12% SDS-PAGE and electro-transferred to PVDF membranes (Immobilon-P; Millipore, Bedford, MA, USA), using a Bio-Rad Semi-Dry Transfer Cell. The blots were then incubated with primary antibodies against α-actinin (ACTN), p53, glucose-regulated protein 78 (GRP78) (Santa Cruz Biotechnology, Dallas, TX, USA), C/EBP homologous protein (CHOP) (Cell Signaling, Danvers, MA, USA), cyclin D1, and p21 (Abcam, Cambridge, UK). Thereafter, the blots were incubated with horseradish peroxidase-conjugated secondary antibody (Santa Cruz Biotechnology). The immunoreactive proteins were detected using ECL^TM^ Western Blotting Detection Reagent and Amersham Hyperfilm^TM^ ECL (GE Healthcare, Waukesha, WA, USA). The procedural details were described in our previous publications [33,34].

### 4.5. Reverse Transcription-Polymerase Chain Reaction (RT-PCR)

Total RNA was isolated from MDA-MB-231, ZR75-1, and MCF-7 cells using TRIsure reagent (Bioline, London, UK). The procedural details are described in our previous publications [35,36]. Reverse transcription for first-strand cDNA synthesis was carried out using MMLV reverse transcriptase (Epicentre Biotechnologies, Madison, WI, USA), with 1 μg of total RNA for 60 min at 37 °C. The PCR reactions were run on a Veriti Thermal Cycler (Applied Biosystems, Waltham, MA, USA). The PCR primers are listed below (Table 1).

### 4.6. Fluorescence-Activated Cell Sorting (FACS), Cell Cycle Profile, Cellular Proliferation, and Cellular Apoptosis Analysis

The cells were fixed in 70% ice-cold ethanol and stored at −30 °C overnight, after which they were washed twice with ice-cold phosphate-buffered saline (PBS) supplemented with 1% FBS and stained with propidium iodide (PI) solution (5 μg/mL PI in PBS, 0.5% Triton X-100, and 0.5 μg/mL RNase A) for 30 min at 37 °C in the dark. The cell cycle distribution was then evaluated using FACS based on the cellular DNA content. Cell proliferation was assessed using FITC-BrdU (5-Bromo-2-deoxyuridine) Flow Kits, according to the manufacturer’s instructions (BD Biosciences). The early and late stages of apoptotic cells were evaluated using a fluorescein Phycoerythrin (PE)-Annexin V Apoptosis Detection Kit (BD Biosciences) according to the manufacturer’s protocol. The cells were stained with PE-Annexin V as well as 7-Amino-Actinomycin (7-AAD) to determine the effects of the indicated drugs (*T. mongolicum* and *T. formosanum*) on early apoptosis (PE-Annexin positive and 7-AAD negative), late apoptosis (PE-Annexin positive and 7-AAD positive), and necrosis (PE-Annexin negative and 7-AAD positive). All samples were analyzed using a FACSCalibur flow cytometer (BD Biosciences). Data were analyzed using Cell Quest Pro software, version 6.1 (BD Biosciences, Franklin Lakes, NJ, USA). The procedural details were described previously [35,36].

### 4.7. Colony Formation and Migration Assays

The colony formation was analyzed as described previously [35,36]. Briefly, MDA-MB-231, ZR75-1, and MCF-7 cells were plated in 6-well plates at a density of 500 cells/well and cultured for 10 days. The colonies were then fixed in methanol and stained with 0.5% crystal violet. The colonies were measured using ImageJ software (National Institutes of Health, Bethesda, MD, USA), and those larger than 0.1 mm in diameter were counted.

To assay cell migration, MDA-MB-231, ZR75-1, and MCF-7 cells were seeded into 12-well plates. When the cell confluence reached 90%, the medium was removed, and a wound was made in the monolayer with a pipette tip. The plate was then washed three times to remove the non-adherent cells. The wound area was photographed immediately after wounding (0 h) and 18 h or 24 h after wounding. The migration rates were computed based on the change in wound area measured with ImageJ software (National Institute of Mental Health, Bethesda, MD, USA).

### 4.8. Mitochondrial Membrane Potential Analysis

Mitochondrial membrane potential was measured using a BD MitoScreen Flow Cytometry Mitochondrial Membrane Potential Detection Kit (BD Biosciences, Franklin Lakes, NJ, USA), according to the manufacturer’s instructions. The procedural details were described in our previous publications [33,34]. All dead and viable cells were harvested, washed with PBS, and incubated with a 1× binding buffer containing the MMP-sensitive fluorescent dye JC-1 (5,5′,6,6′-tetrachloro-1,1′,3,3′-tetraethylbenzimi-dazolyl carbo-cyanine iodide) for 15 min at 37 °C in the dark. The cells were then washed, and JC-1 fluorescence was measured in a flow cytometer with excitation at 488 nm and emission at 530 nm (FL1-H channel for green fluorescence) for the monomer and 580 nm (FL2-H channel for red fluorescence) for aggregates, with a FACSCalibur flow cytometer using Cell Quest Pro software (BD Biosciences, Franklin Lakes, NJ, USA). The mitochondrial depolarization was measured as a function of the decrease in the red/green fluorescence intensity ratio.

### 4.9. Detection of the Oxygen Consumption Rate (OCR) and Extracellular Acidification Rate (ECAR)

The cellular OCR and ECAR were measured using a Seahorse XF24 bioenergetic assay, according to the manufacturer’s instructions (Agilent, Santa Clara, CA, USA). The procedural details were described in our previous publications [36,37]. Briefly, MDA-MB-231 cells (5 × 10^3^ cells/well) were plated on an XF24 microplate and cultured for 3 days. Thereafter, XF24 bioenergetic assays were started by replacing the exhausted medium with sodium-bicarbonate-free DMEM (pH 7.4), supplemented with 2% FBS and 2% horse serum. The OCR and ECAR were measured at a steady state, and then oligomycin (1 μM), carbonyl cyanide 4-(trifluoromethoxy)phenylhydrazone (FCCP; 0.5 μM), and rotenone/antimycin (0.5 μM) were added sequentially to the wells to obtain values for the maximal and non-mitochondrial respiration rates.

### 4.10. Statistical Analysis 

The values are expressed as the mean ±SD of at least three independent experiments. All comparisons between groups were made using unpaired two-tailed *t*-tests. The statistical significance was set at *p* < 0.05.

## 5. Conclusions

Our current findings demonstrate that *T. mongolicum* showed greater cytotoxicity against all three tested breast cancer cell lines, especially the TNBC MDA-MB-231 cell line. The cytotoxicity was mediated through the induction of apoptosis, the reduction of cell proliferation, the disruption of the mitochondrial membrane potential, and/or the downregulation of the oxygen consumption rate. Our *Taraxacum* studies using aqueous extracts can be assumed to parallel its traditional consumption, i.e., fed ad libitum with infusions. In addition to the saving of time and money for isolation of bioactive compounds, the beneficial evidence of various *Taraxacum* extracts provides the choice of future combinatory therapy with current TNBC therapy.

## Figures and Tables

**Table 1:** PCR primers were used in this study.

Primer Name	Sequence (5′ → 3′)
*p21*	Forward: 5′-CTGAGCCGCGACTGTGATGCG-3′ Reverse: 5′-GGTCTGCCGCCGTTTTCGACC-3′
*β-actin*	Forward: 5′-GTGGGGCGCCCCAGGCACCA-3′ Reverse: 5′-CTCCTTAATGTCACGCACGATTTC-3′
*GAPDH*	Forward: 5′-CTTCATTGACCTCAACTAC-3′ Reverse: 5’-GCCATCCACAGTCTTCTG-3′
*18S*	Forward: 5′-CAGCCACCCGAGATTGAGCA-3′ Reverse: 5′-TAGTAGCGACGGGCGGTGTG-3′

## Data Availability

Not applicable.
